# Polymorphism and orientation control of copper-dicarboxylate metal–organic framework thin films through vapour- and liquid-phase growth†

**DOI:** 10.1039/d3ce01296d

**Published:** 2024-02-02

**Authors:** Víctor Rubio-Giménez, Francesco Carraro, Sebastian Hofer, Mario Fratschko, Timothée Stassin, Sabina Rodríguez-Hermida, Benedikt Schrode, Luisa Barba, Roland Resel, Paolo Falcaro, Rob Ameloot

**Affiliations:** a Centre for Membrane Separations, Adsorption, Catalysis and Spectroscopy (cMACS), KU Leuven Celestijnenlaan 200F 3001 Leuven Belgium victorrubio.gimenez@kuleuven.be rob.ameloot@kuleuven.be; b Institute of Physical and Theoretical Chemistry, Graz University of Technology Stremayrgasse 9/Z2 8010 Graz Austria paolo.falcaro@tugraz.at; c Institute of Solid State Physics, Graz University of Technology Petersgasse 16 8010 Graz Austria roland.resel@tugraz.at; d Istituto di Cristallografia – Sincrotrone Elettra, Consiglio Nazionale delle Ricerche Area Science Park 34142 Basovizza Italy

## Abstract

Precise control over the crystalline phase and crystallographic orientation within thin films of metal–organic frameworks (MOFs) is highly desirable. Here, we report a comparison of the liquid- and vapour-phase film deposition of two copper-dicarboxylate MOFs starting from an oriented metal hydroxide precursor. X-ray diffraction revealed that the vapour- or liquid-phase reaction of the linker with this precursor results in different crystalline phases, morphologies, and orientations. Pole figure analysis showed that solution-based growth of the MOFs follows the axial texture of the metal hydroxide precursor, resulting in heteroepitaxy. In contrast, the vapour-phase method results in non-epitaxial growth with uniplanar texture only.

## Abstract 2

Liquid- and vapour-phase conversion of aligned Cu(OH)_2_ nanobelts into crystalline coatings of Cu-BDC and Cu-CDC MOFs, leads to differences in the crystal phase, morphology and crystallographic orientation.

## Graphical abstract

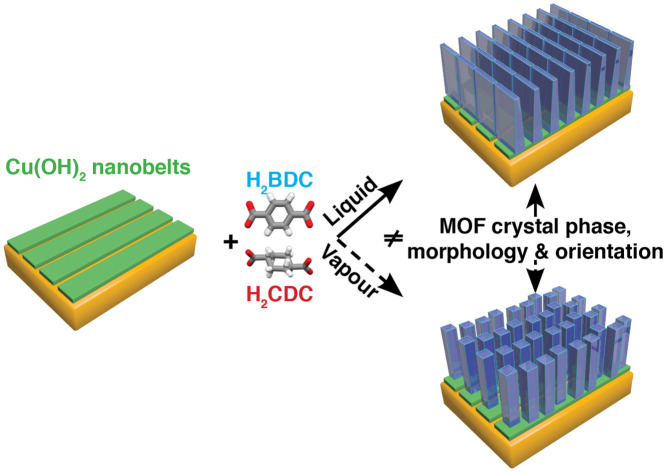

Metal–organic frameworks (MOFs) are extended materials composed of inorganic metal ions or clusters and organic linkers. Researchers have designed MOF materials with exceptional properties for gas storage,^1–3^ separation,^4,5^ and catalysis.^6,7^ For other applications, such as microelectronics,^8–10^ MOF materials are needed in thin-film format. In addition, control over crystallite morphology and crystallographic orientation would be desired to influence pore accessibility and anisotropic stimuli-responsive properties.^8,11^ Yet, the strategies to control the orientation of MOF crystalline coatings have received limited attention.^12^

Liquid-phase growth using substrates functionalized with self-assembled monolayers has had great success in controlling the crystallographic orientation in various MOF thin films.^13^ However, while several studies illustrated control over the out-of-plane crystallographic orientation, the in-plane orientation has only been addressed for a handful of MOF thin films.^12^ Falcaro, Takahashi, and co-workers pioneered a heteroepitaxial method yielding centimetre-scale MOF films oriented both in-plane and out-of-plane.^14^ The careful selection of an oriented and crystalline metal hydroxide precursor with lattice parameters compatible with those of the target MOF resulted in MOF crystallites that inherited the orientation of the precursor. Following these principles, various Cu-carboxylate MOFs have been successfully grown on oriented Cu(OH)_2_ nanobelts and nanotubes,^14–19^ including MOF-on-MOF heterostructures.^20^ This has resulted in oriented MOF coatings and micropatterns with anisotropic functional properties such as fluorescence, plasmonic resonance, nonlinear optics and other guest-depended properties.^19,21–23^

Thus far, all instances of MOF heteroepitaxial growth relied on the solid–liquid reaction of a crystalline metal hydroxide precursor with a linker solution. However, in some cases, MOF films can also form at the solid–vapour interface by reacting vapourised linkers with metal, oxide or hydroxide precursors in a chemical vapour deposition (CVD) process. This MOF-CVD vapour-phase process enables solvent-free fabrication of crystalline MOF thin films with controllable thickness and is compatible with microfabrication standards.^24–26^ A myriad of MOFs and coordination polymers have been synthesized under these solvent-free conditions,^26^ including various Cu-carboxylate MOFs.^27–31^ Even though MOF films oriented in the out-of-plane direction have been obtained *via* vapour-phase conversion,^28,32,33^ in-plane oriented MOF films or heteroepitaxial growth have been just barely explored *via* vapour-phase syntheses. Besides, these solvent-free methods can also yield unique MOF crystalline phases, not accessible in solution.^31,34^

Here, we compare liquid- and vapour-phase conditions to grow two Cu-dicarboxylate MOFs from a precursor film of oriented Cu(OH)_2_ nanobelts. We selected ligands that can afford heteroepitaxial matching conditions, namely BDC (1,4-benzenedicarboxylate) and CDC (*trans*-1,4-cyclohexanedicarboxylate). Remarkably, the variety of coordination modes for Cu^II^ and the different arrangements of BDC and CDC dicarboxylate linkers have resulted in various polymorphs of both Cu-BDC^35–38^ and Cu-CDC.^39–42^

Both the liquid- and vapour-phase conversion methods start with the deposition of oriented Cu(OH)_2_ nanobelts onto Si substrates.^14,17^ Grazing incidence X-ray diffraction (GIXRD) pole figure analysis confirms the axial texture of the coated substrates:^43^ the Cu(OH)_2_ nanobelts are aligned parallel to the substrate and their crystallographic *a*-axes all point in the same direction (Fig. 1, S1 and S2†). For liquid-phase conversion, these samples were placed in a H_2_O/EtOH solution of H_2_BDC or H_2_CDC (0.1 mg mL^−1^, 30 minutes, room temperature, Fig. S3a†), followed by rinsing with pure EtOH and drying in air. For the vapour-phase procedure (Fig. S3b†), the Cu(OH)_2_-nanobelt-coated substrates were placed in a Schlenk tube with an excess of linker in a separate boat. The tube was then evacuated (∼10^−1^ mbar) and heated at 200 °C for 16 hours. The Cu(OH)_2_ conversion to Cu-BDC and Cu-CDC was examined by scanning electron microscopy (SEM) and synchrotron GIXRD.

**Fig. 1:**
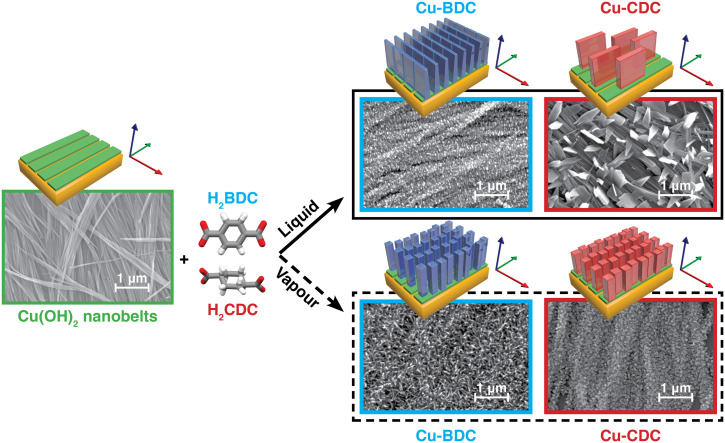
Liquid- and vapour-phase conversion of aligned Cu(OH)_2_ nanobelts into Cu-BDC and Cu-CDC MOFs. The resulting MOFs have a different crystal phase, morphology, and crystallographic orientation. Top-view SEM images of Cu(OH)_2_ nanobelts before (green) and after conversion into Cu-BDC (blue) and Cu-CDC (red) under liquid- (solid line box) and vapour-phase (dashed line box) conditions.

The liquid-phase conversion of Cu(OH)_2_ nanobelts to Cu-BDC yielded a continuous film of oriented crystallites growing orthogonally to the nanobelts (Fig. 1), thus confirming previous reports.^14^ The dense MOF coating is composed of platelet-shaped crystallites of approximately 50 nm in height (Fig. 1 and S4b†). Conversely, the Cu-BDC layer resulting from vapour-phase conversion was formed by densely packed needle-shaped crystallites of *ca.* 250 × 25 nm^2^ (Fig. 1 and S4c†), which appear to have a seemingly random orientation with respect to the nanobelt precursors. For Cu-CDC, liquid-phase conversion yielded sparse and considerably larger platelet-like crystallites oriented orthogonally with respect to the nanobelts. In contrast, vapour-phase reaction conditions resulted in a densely packed film of ∼20 nm block-shaped crystallites with similar morphology to those obtained from the conversion of CuO films under equivalent vapour-phase conditions.^28^ Morphological details are reported in Fig. 1 and S4d and e.†

The phase and crystalline texture of the films were subsequently examined by synchrotron GIXRD. Reciprocal space maps (RSMs, Fig. S5†) and 1-dimensional diffraction patterns (obtained from the complete q space integration of the RSMs, Fig. 2) were processed from pixel images using the GIDVis software package.^44^ Subsequently, the diffractograms were compared to known phases of Cu-BDC and Cu-CDC (Table S1†). As shown in Fig. 2, the vapour-phase reactions with H_2_BDC or H_2_CDC produced phase-pure crystalline materials that match previously reported crystal structures: ZUBKEO^37^ for Cu-BDC and SIWGUB^42^ for Cu-CDC. This observation is in agreement with the thin films obtained from non-textured Cu and CuO precursor layers under equivalent vapour-phase conditions.^28^ In contrast, the liquid-phase conditions generated different crystalline phases for both Cu-BDC and Cu-CDC, which could not be assigned to any solved crystal phases, but are compatible with the structure originally reported by Falcaro, Takahashi, and co-workers.^14^

**Fig. 2:**
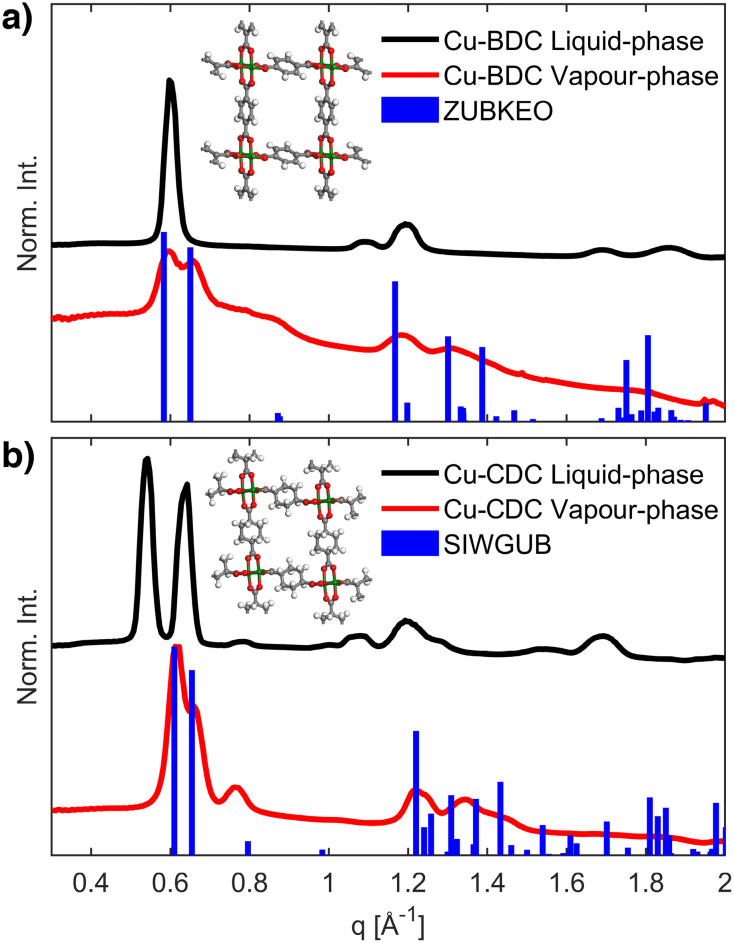
GIXRD patterns of Cu-BDC (a) and Cu-CDC (b) films grown from Cu(OH)_2_ nanobelts. In both cases, the diffractograms simulated for known crystal phases (blue) match only with the materials produced *via* vapour-phase synthesis (red) and not with those resulting from the liquid-phase procedure (black). The inset shows the ZUBKEO (a)^37^ and SIWGUB (b)^42^ crystal structures. Cu, O, C, and H atoms are coloured green, red, grey, and white, respectively.

To qualitatively determine the degree of heteroepitaxial growth under both liquid and vapour-phase conditions, we analysed the in-plane and out-of-plane crystalline orientation of the Cu-BDC and Cu-CDC films. All samples showed preferential orientation, at least in the out-of-plane direction. For the samples obtained under vapour-phase conditions, this is shown in Fig. 3 by a comparison of the RSMs with the peak positions calculated for the ZUBKEO^37^ and SIWGUB^42^ phases for Cu-BDC and Cu-CDC, respectively. Although incomplete arcs rather than defined spots are observed, we can assign a moderate out-of-plane (100) orientation since the calculated positions are positioned at the centre of the experimental arc-like diffraction features. The (100) orientation indicates that for both MOF phases, the pores are generally oriented perpendicular to the Si substrate (Fig. S6†), which is ideal for guest accessibility. This result is in agreement with the thin films produced under equivalent vapour-phase conditions from Cu and CuO precursor layers.^28^ Cu-BDC and Cu-CDC thin films fabricated *via* liquid-phase conversion also show moderate out-of-plane orientations with respect to the substrate, identified by enhanced intensities around *q*_*xy*_ = 0 Å^−1^ (compare Fig. S5a and b†).^36^ In the case of Cu-BDC, a preferred out-of-plane orientation has already been observed.^14^

**Fig. 3:**
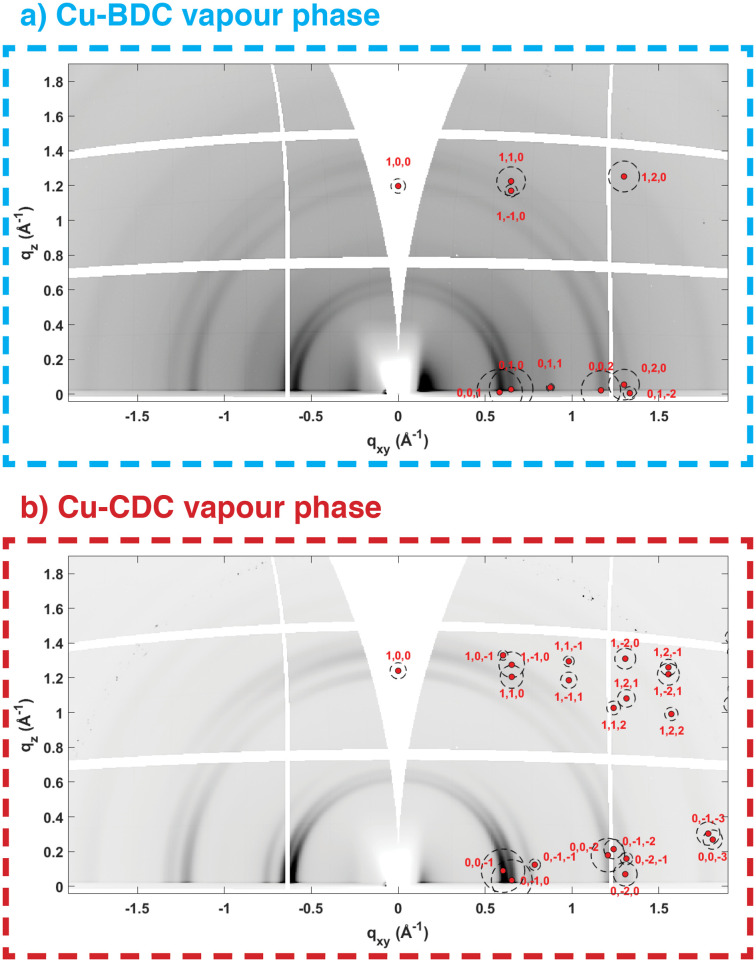
Reciprocal space maps of Cu-BDC (a) and of Cu-CDC (b) prepared by vapour conversion together with indexing of the diffraction features with the known phases of ZUBKEO^37^ and SIWGUB^42^ for a (100) crystallite orientation overlaid on the positive *q*_*xy*_ side of the maps. Red points and the centre of circles give the expected positions of the diffraction peaks, and the areas inside the circles give the square of the structure factors, which are proportional to the expected intensities.

The in-plane crystallographic orientation can be qualitatively assessed through the pole figure representation of the rotating GIXRD experiments depicted in Fig. S7a.† A series of detector pixel images with changing *φ* angles (0–360°) were recorded by rotating the film surface plane around its *z*-axis (Fig. S7a†). These images were converted into q space and processed using the GIDVis software to create pole figures (Fig. S7b†).^44^ A single pole figure gives the spatial orientation of a specific Bragg peak; combining a number of pole figures reveals the distribution of crystallographic orientations within the samples. Pole figure generation does not require knowledge of the crystal structure (or the crystal lattice). In the case of Cu-BDC films obtained through liquid-phase reaction, we present the pole figures of the Bragg peaks at 0.595 Å^−1^ and 1.088 Å^−1^ in Fig. 4a. The characteristic distributions of enhanced pole densities within both pole figures allow to assign the same axial texture to the distribution of the crystallites as for the underlying nanobelt precursor (Fig. S2†). The featured axes of the Cu(OH)_2_ nanobelts and the MOF crystals coincide. This experiment fully confirms the claimed heteroepitaxy for the Cu-BDC system on Cu(OH)_2_ nanobelts.^14^ A similar situation is found for Cu-CDC obtained from solution synthesis (Fig. 4b): an axial texture is revealed that is related to the crystal texture of the nanobelt precursor substrate. While the liquid-phase conversion of Cu(OH)_2_ nanobelt precursors leads to epitaxial order for both MOFs, the pole figures for vapour-converted samples show no pronounced variation in intensity along any in-plane direction (Fig. 4c and d). Therefore, despite the use of Cu(OH)_2_ nanobelts, the vapour-phase conversion protocol does not lead to significant in-plane epitaxial order of the final MOF crystallites. Nevertheless, the out-of-plane orientation of the crystallites is visible through the reduced intensity at the centre of the pole figures. Note that experimental artifacts are visible in Fig. 4c due to a small quadratic sample size (four-fold symmetry) and blind regions of the detector (white circles).

**Fig. 4:**
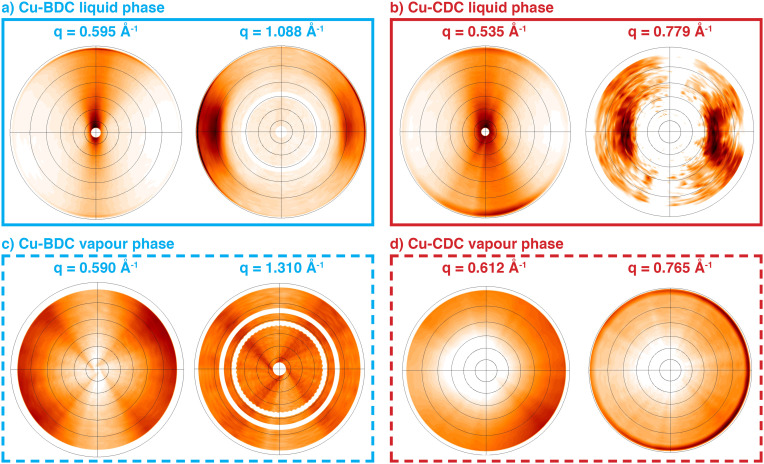
Pole figures for a) liquid-phase Cu-BDC recorded at *q* = 0.595 Å^−1^ and 1.088 Å^−1^; b) liquid-phase Cu-CDC recorded at *q* = 0.535 Å^−1^ and at *q* = 0.779 Å^−1^; c) vapour-phase Cu-BDC recorded at *q* = 0.590 Å^−1^ (for the 001 Bragg peak) and 1.310 Å^−1^ (020); and of d) Cu-CDC recorded at *q* = 0.612 Å^−1^ (001) and 0.765 Å^−1^ (011).

## Conclusions

In conclusion, we showed that two copper-carboxylate MOFs could be grown by reacting textured Cu(OH)_2_ nanobelt precursors with their respective linkers in the vapour and liquid phase. These different synthetic conditions resulted in MOF films with different crystalline phases, crystallographic orientations, and morphologies. While both liquid- and vapour-phase growth induce out-of-plane orientation, only liquid-phase conditions achieve heteroepitaxial MOF growth. We hypothesize that the absence of solvent, together with the higher ligand concentrations and reaction temperature in the vapour-phase synthesis, contributes to the crystallization of different polymorph of Cu-BDC and Cu-CDC and prevents heteroepitaxial growth. These results highlight the importance of appropriately selecting the growth method of MOF layers in view of fabricating functional devices.

## Author contributions

Víctor Rubio-Giménez: investigation, writing – original draft, writing review & editing; Francesco Carraro: investigation, methodology, writing review & editing; Sebastian Hofer: investigation, formal analysis, writing review & editing; Mario Fratschko: investigation, formal analysis; Timothée Stassin: investigation, methodology; Sabina Rodríguez-Hermida: investigation, methodology; Benedikt Schrode: investigation, formal analysis, software; Luisa Barba: investigation; Roland Resel: conceptualization, supervision, funding acquisition, writing review & editing; Paolo Falcaro: conceptualization, supervision, funding acquisition, writing review & editing; Rob Ameloot: conceptualization, supervision, funding acquisition, writing review & editing.

## Conflicts of interest

There are no conflicts to declare.

## Supplementary Material

CE-026-D3CE01296D-s001
