# Supplementary material for: Polymorphism and orientation control of copper-dicarboxylate metal–organic framework thin films through vapour- and liquid-phase growth
Source: CrystEngComm. 2024 Feb 2;26(8):1071–6. doi: 10.1039/d3ce01296d (PMC10877460; doi:10.1039/d3ce01296d)
Supplement: CE-026-D3CE01296D-s001 [file CE-026-D3CE01296D-s001.pdf]

Electronic Supplementary Material (ESI) for CrystEngComm.
This journal is © The Royal Society of Chemistry 2024

Supplementary Information

Polymorphism and orientation control of copper-dicarboxylate metal-organic framework thin films through vapour- and liquid-phase growth

Víctor Rubio-Giménez,[a,‡,*] Francesco Carraro,[b,‡] Sebastian Hofer,[c] Mario Fratschko,[c] Timothée Stassin,[a] Sabina Rodríguez-Hermida,[a] Benedikt Schrode,[c] Luisa Barba,[d] Roland Resel[c,*] Paolo Falcaro[b,*] and Rob Ameloot[a,*]

a Centre for Membrane Separations, Adsorption, Catalysis and Spectroscopy (cMACS), KU Leuven, Celestijnenlaan 200F box 2454, 3001 Leuven, Belgium.

b Institute of Solid State Physics, Graz University of Technology, Petersgasse 16, 8010 Graz, Austria.

c Institute of Physical and Theoretical Chemistry, Graz University of Technology, Stremayrgasse 9/Z2, 8010 Graz, Austria.

d Istituto di Cristallografia – Sincrotrone Elettra, Consiglio Nazionale delle Ricerche, Area Science Park, 34142 Basovizza, Italy.

Table of contents

SI1. Materials, reagents and substrates 2
SI2. Synthetic details 2
Synthesis of oriented $Cu(OH)_2$ nanobelts 2
Liquid phase conversion of $Cu(OH)_2$ nanobelts to Cu-CDC or Cu-BDC 2
Vapour-phase conversion of $Cu(OH)_2$ nanobelts to Cu-CDC or Cu-BDC 2
SI1. Physical characterization 2
Grazing incidence X-ray diffraction (GIXRD) 2
Scanning electron microscopy (SEM) 3
SI5. Results and Discussion 4
Figure S1. Crystalline structure of the $Cu(OH)_2$ nanobelts 4
Figure S2. Pole figures of $Cu(OH)_2$ nanobelt substrates 5
Figure S3. Schematic illustrations of the liquid- and vapour-phase conversion procedures and reaction set-ups 6
Figure S4. Additional top-view SEM images 7
Figure S5. Reciprocal space maps obtained from synchrotron GIXRD data 8
Table S1. Previously reported Cu-BDC and Cu-CDC crystalline structures 8
Figure S6. Crystalline structures of vapour converted Cu-BDC and Cu-CDC onto $Cu(OH)_2$ nanobelts 9
Figure S7. Geometry of a rotating GIXRD experiment 10
SI6. References 11

SI2. Materials, reagents, and substrates

All reagents and solvents were used as received without further purification. Terephthalic acid or 1,4-benzenedicarboxylic acid (H_2BDC, >99 %) and trans-1,4-cyclohexanedicarboxylic acid (H_2CDC, >97 %) were purchased from TCI Europe. Copper(II) sulfate pentahydrate ($CuSO_4{\cdot}5H_2O$, >98%) was purchased from Sigma-Aldrich.

Single-side-polished, back-etched, p-type Si wafers were purchased from Si-Mat. Appropriately sized substrates (~15x15 mm) were then hand-cleaved from these 150 mm diameter Si wafers.

SI3. Synthetic details

Synthesis of oriented $Cu(OH)_2$ nanobelts: Si substrates coated with $Cu(OH)_2$ nanobelts were prepared using a previously described protocol with slight modifications.[1] First, 202 mg of $CuSO_4{\cdot}5H_2O$ were dissolved in 20 ml of water. To this solution, 6 mL of a 150 mM ammonia aqueous solution and 1.2 mL of a 50% w/v NaOH solution were added dropwise. The mixture was stirred at room temperature for 1 h and then left in an oven at 40°C for 30 minutes. Next, $Cu(OH)_2$ nanobelts were centrifuged, washed twice with water, twice with ethanol, and finally re-dispersed in 10 mL of ethanol. This suspension was then spread on the surface of a water bath from one side of a rectangular basin using a syringe pump. Finally, the oriented $Cu(OH)_2$ nanobelts floating film was manually transferred from the water surface to 15x15 mm Si substrates, which were subsequently rinsed with ethanol and dried by blowing air. The process of forming the floating film of oriented $Cu(OH)_2$ nanobelts and transferring it to a substrate is described in detail in reference 1, which includes a schematic illustration of the procedure in the SI.

Liquid phase conversion of $Cu(OH)_2$ nanobelts to Cu-CDC or Cu-BDC: Si substrates coated with $Cu(OH)_2$ nanobelts were immersed in a saturated ligand solution (2.86 ml of water, 7.14 ml of ethanol mixture, 0.1 g of H_2BDC or H_2CDC). After 30 min, the substrates were removed from the solution, washed with ethanol, and dried under air.

Vapour phase conversion of $Cu(OH)_2$ nanobelts to Cu-CDC or Cu-BDC: Si substrates coated with $Cu(OH)_2$ nanobelts were placed in a 250 mL Schlenk tube together with a glass vial containing 500 mg of H_2CDC or H_2BDC ligand. Next, the tube was evacuated under dynamic vacuum (~10^{-2} mbar) for 20 min. The closed, evacuated tube was then placed in a forced convection oven preheated to 200 °C for 16 hours. Afterwards, the tube was removed from the oven and vented. The samples were quickly removed from the tube while hot and placed in the oven again at 200 °C for 30 additional minutes.

SI4. Physical characterization

Grazing incidence X-ray diffraction (GIXRD) measurements were performed at the XRD1 beamline of the Elettra synchrotron (Trieste, Italy) using a stationary Pilatus 2M detector approximately 200 mm from the sample. Data were collected at a wavelength of 1.4 Å and an incident angle of approximately 0.5°. In order to investigate a larger volume of reciprocal space, the samples were rotated during the measurements (around the sample surface normal), and diffraction patterns were recorded in angular steps integrating 2 degrees (Figure S6a). All diffraction data were converted from pixel patterns to reciprocal space (Figure S6b). Pole figures were calculated by using all detector images. Reciprocal space maps are obtained by integration of the detected intensities of each pixel for a complete sample rotation so that the result can be presented as a function of the in-plane and out-of-plane parts of the scattering vector, q_{xy} and q_z, respectively. Please note that $q_{xy}^2 = q_x^2 + q_y^2$. 1D diffractograms were obtained by

integrating the reciprocal space maps in terms of the length of the scatting vector. The data treatment was performed using GIDVis software.[2]

Scanning electron microscopy (SEM) images were acquired using a Philips XL30 FEG SEM microscope operating at an accelerating voltage of 10 keV. Samples were cleaved and sputter-coated with 2 nm of Pt before inspection using a Quorum Q150 sputter coater.

SI5. Results and Discussion

Figure S1. Crystal structure of the $Cu(OH)_2$ nanobelts with orthorhombic symmetry and lattice constants a = 2.947 Å, b = 10.593 Å and c = 5.2564 Å^3 with the crystallographic a–axis along the long axes of the nanobelts.

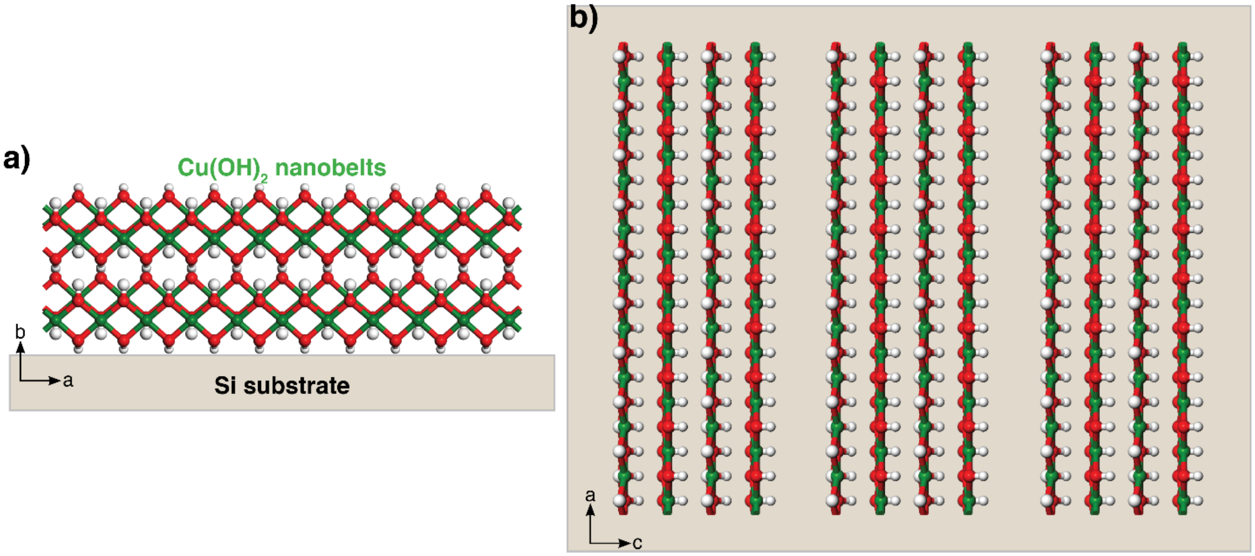

Figure S2. Pole figures of $Cu(OH)_2$ nanobelt substrates recorded at q = 1.19 Å^{-1} (a) for the 020 peak and at 2.52 Å^{-1} (b) for the 111 peak. (c) Stereographic projection of $Cu(OH)_2$ crystals according to an axial texture: crystals with the crystallographic *a*–axis along the substrate surface (these directions are represented by full black circles); an in-plane mosaicity of 10° is considered.

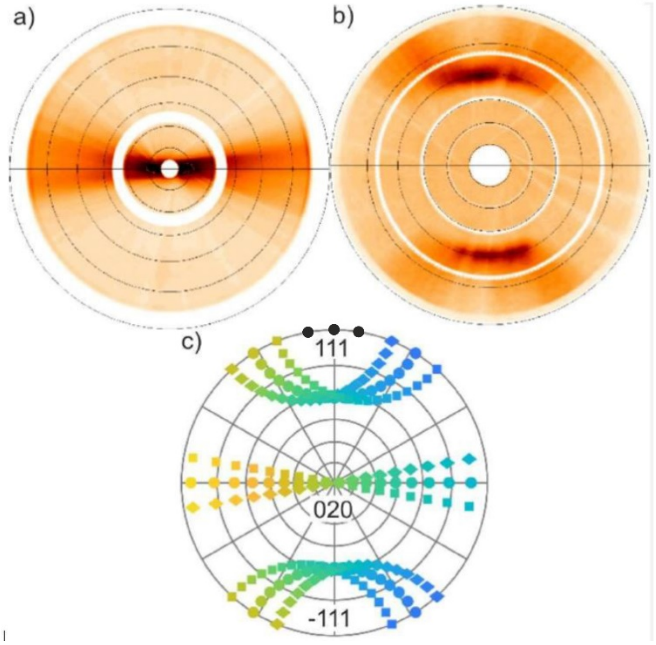

Figure S3. Schematic illustrations of the liquid- (a) and vapour-phase (b) conversion procedures and reaction set-ups. The liquid-phase conversion was performed by immersing Si substrates coated with $Cu(OH)_2$ nanobelts in a saturated ligand solution for 30 min. For the vapour-phase conversion Si substrates coated with $Cu(OH)_2$ nanobelts are positioned approximately in the middle of a 100 mL Schlenk tube together with linker powder in an open glass boat at the opposite end. After evacuating under dynamic vacuum (~10^{-2} mbar) for 20 min, the evacuated tube stopcock is closed and placed in a forced convection oven preheated at 200 °C for 16 hours. Created with BioRender.com

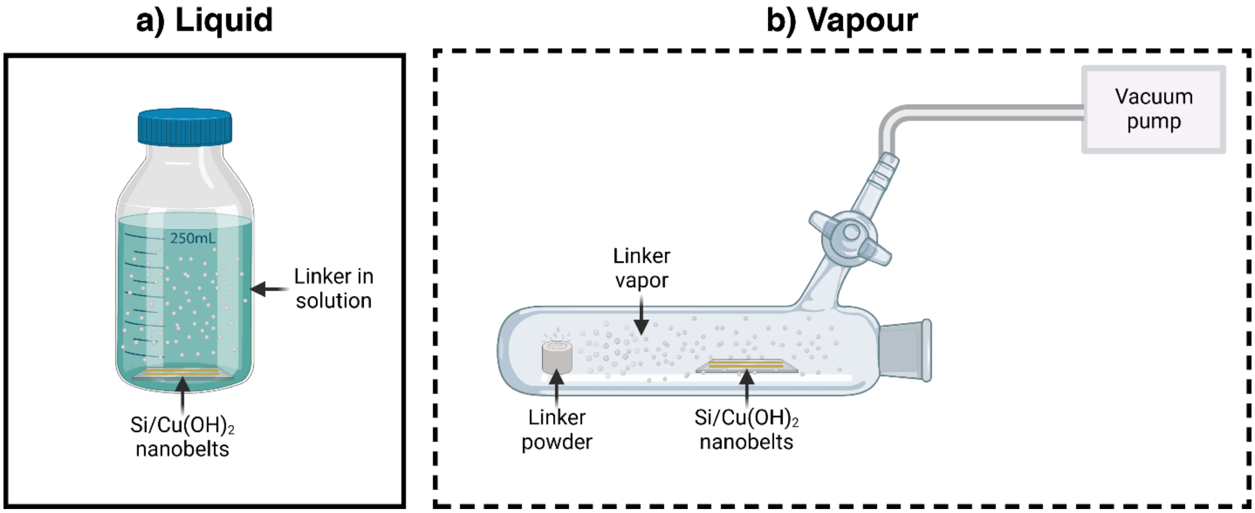

Figure S4. Additional top-view SEM images of a) aligned $Cu(OH)_2$ nanobelts deposited on a Si substrate; b) Cu-BDC crystalline film converted under liquid-phase conditions c) Cu-BDC crystalline film converted under vapour-phase conditions; d) Cu-CDC crystalline film converted under liquid-phase conditions; e) Cu-CDC crystalline film converted under vapour-phase conditions.

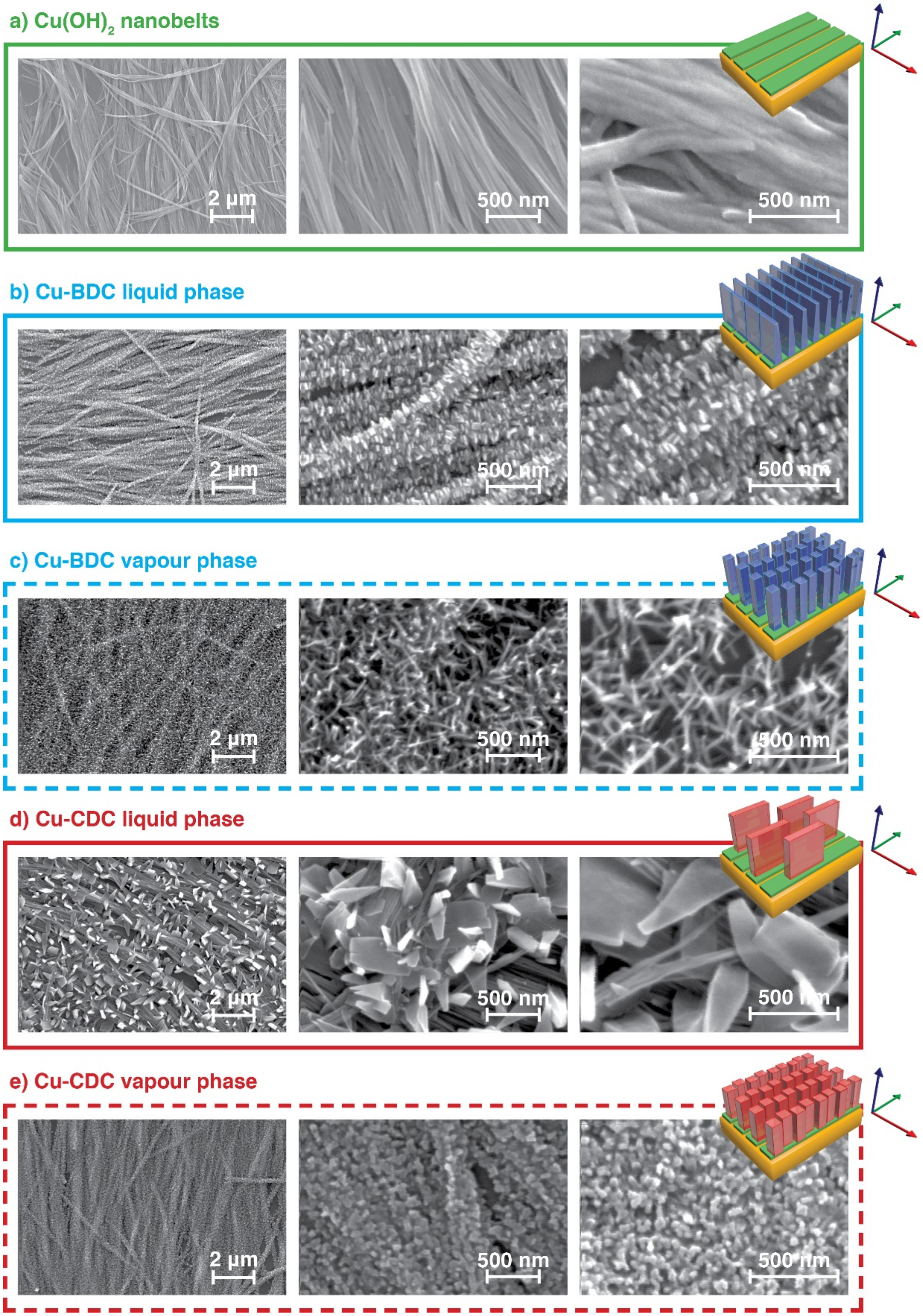

Figure S5. Reciprocal space maps obtained from synchrotron GIXRD data for a) Cu-BDC crystalline film converted under liquid-phase conditions b) Cu-BDC crystalline film converted under vapour-phase conditions; c) Cu-CDC crystalline film converted under liquid-phase conditions; d) Cu-CDC crystalline film converted under vapour-phase conditions.

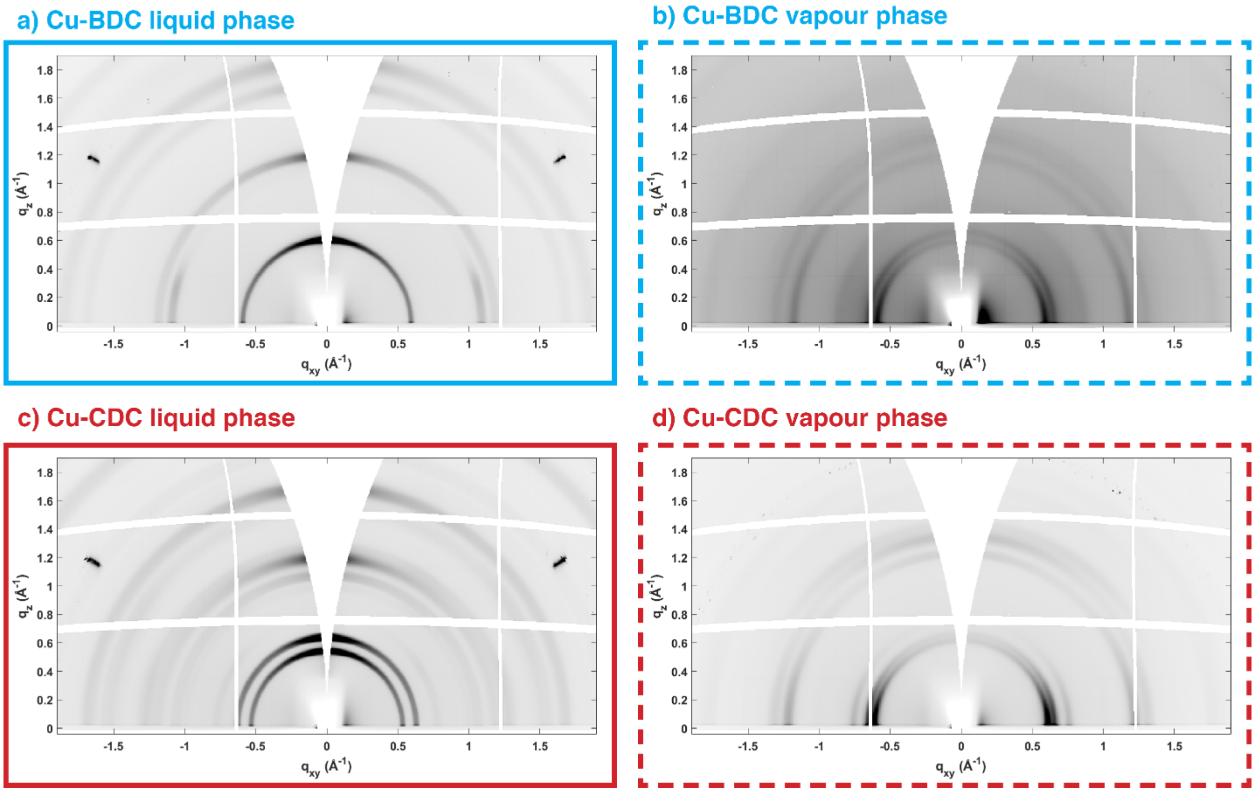

Table S1. Previously reported Cu-BDC and Cu-CDC crystalline structures, detailing lattice parameters and data from the CCDC database, Cambridge, UK.

	CCDC code	Space group	a (Å)	b (Å)	c (Å)	α (°)	β (°)	□ (°)	Reference
Cu-BDC ZUBKEO	1056985	P-1	5.2502	9.66899	10.76792	90.291	91.059	92.413	[4]
Cu-BDC PUYREH	687690	C 2/m	11.4143(3)	14.2687(4)	7.7800(2)	90	108.119(1)	90	[5]
Cu-BDC SURMOF-2	-	P4	10.61	5.8	10.61	90	90	90	[6]
Cu-CDC SIWGUB	269992	P-1	5.140(2)	9.856(5)	10.673(5)	77.310(9)	80.697(6)	85.024(9)	[7]

Figure S6. Crystalline structures of vapour converted (a, b) Cu-BDC and (c, d) Cu-CDC (ZUBKEO[4] and SIWGUB,[7] respectively) onto $Cu(OH)_2$ nanobelts, showcasing the (100) orientation with respect to the substrate and the nanobelts. Thus, in both cases, the pore channels lay perpendicular to the surface. Crystallographic axes correspond to the MOF structure only.

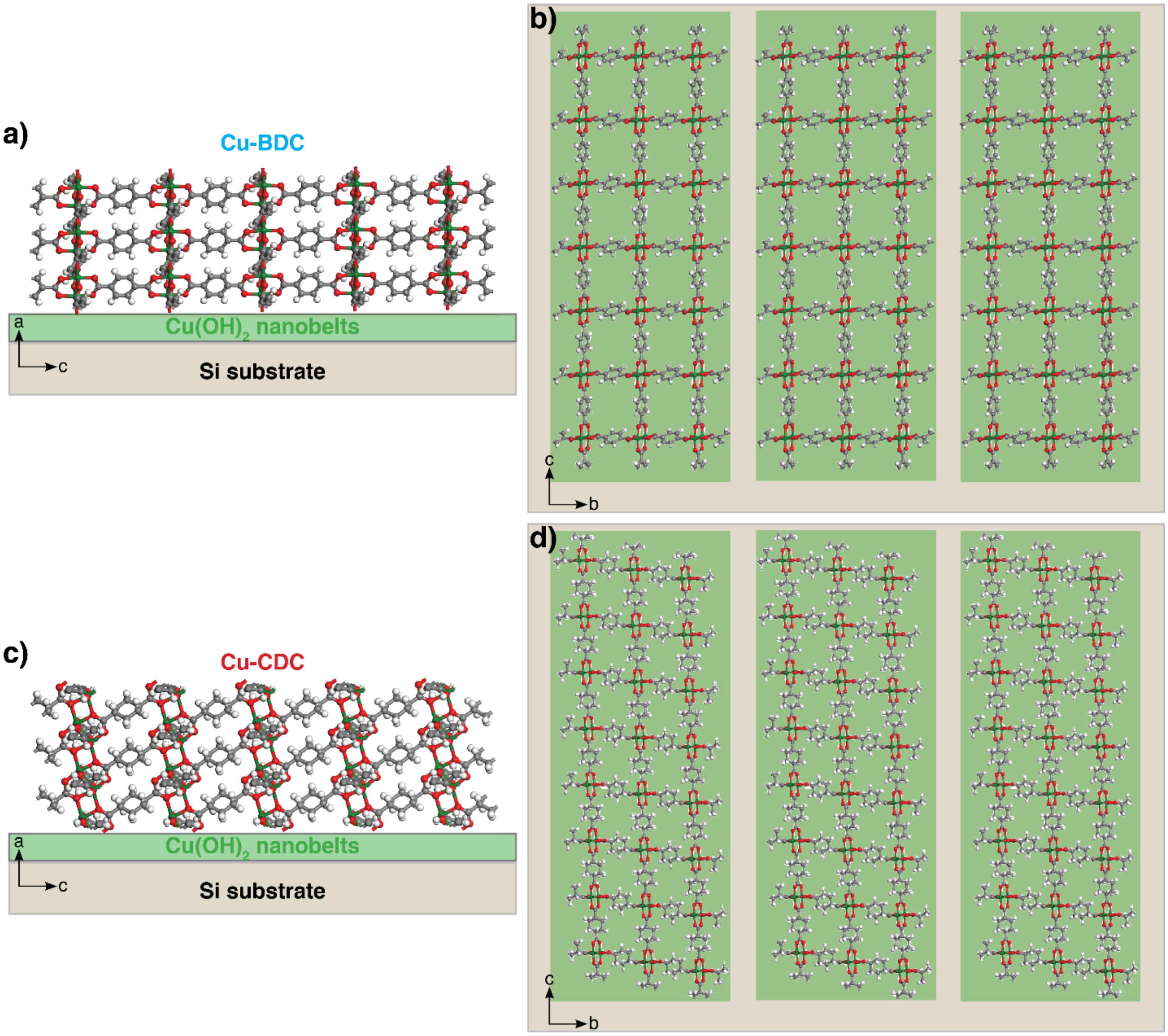

Figure S7. a) Geometry of a rotating GIXRD experiment with k_0 and k as the wavevectors of the primary and the diffracted X-ray beam, respectively. The beams enclose an angle of $\square_i$ and $\square_f$ relative to the substrate surface. The resulting scattering vector q ($\mathbf{q} = \mathbf{k} - \mathbf{k_0}$) is separated in two in-plane components (q_x, q_y) and in an out-of-plane component (q_z). The sample is rotated during the measurement around the surface normal, and a diffraction pattern is taken by integrating a certain range of φ rotation. b) Two detector images measured at different φ angles converted into reciprocal space.

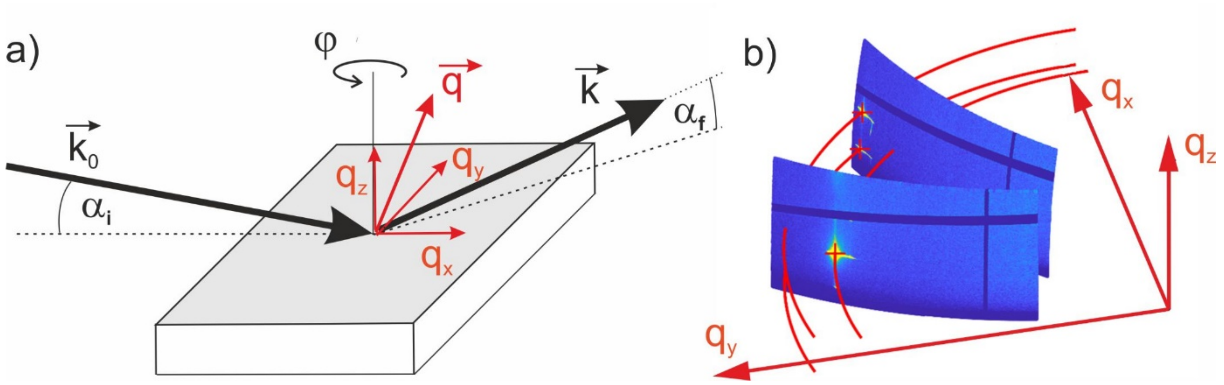

SI6. References

1 P. Falcaro, K. Okada, T. Hara, K. Ikigaki, Y. Tokudome, A. W. Thornton, A. J. Hill, T. Williams, C. Doonan and M. Takahashi, *Nat. Mater.*, 2016, **16**, 342–348.

2 B. Schrode, S. Pachmajer, M. Dohr, C. Röthel, J. Domke, T. Fritz, R. Resel and O. Werzer, *J. Appl. Crystallogr.*, 2019, **52**, 683–689.

3 H. R. Oswald, A. Reller, H. W. Schmalle and E. Dubler, *Acta Crystallogr. Sect. C*, 1990, **46**, 2279–2284.

4 C. G. Carson, G. Brunnello, S. G. Lee, S. S. Jang, R. A. Gerhardt and R. Tannenbaum, *Eur. J. Inorg. Chem.*, 2014, **2014**, 2140–2145.

5 C. G. Carson, K. Hardcastle, J. Schwartz, X. Liu, C. Hoffmann, R. A. Gerhardt and R. Tannenbaum, *Eur. J. Inorg. Chem.*, 2009, **2009**, 2338–2343.

6 H. K. Arslan, O. Shekhah, D. C. F. Wieland, M. Paulus, C. Sternemann, M. A. Schroer, S. Tiemeyer, M. Tolan, R. A. Fischer and C. Wöll, *J. Am. Chem. Soc.*, 2011, **133**, 8158–8161.

7 H. Kumagai, M. Akita-Tanaka, K. Inoue, K. Takahashi, H. Kobayashi, S. Vilminot and M. Kurmoo, *Inorg. Chem.*, 2007, **46**, 5949–5956.
